# Perioperative Ängste und die Angst vor dem Tod

**DOI:** 10.1007/s00101-023-01267-3

**Published:** 2023-03-10

**Authors:** Paraskevi Mavrogiorgou, Hannah Zogas, Georgios Zogas, Georg Juckel, Jan-Florian Heuer

**Affiliations:** 1 Klinik für Psychiatrie Psychotherapie und Präventivmedizin, LWL-Universitätsklinikum, Ruhr Universität Bochum, Alexandrinenstr. 1, 44791 Bochum, Deutschland; 2 Klinik für Anästhesiologie, Intensivmedizin, Notfallmedizin und Schmerztherapie (AINS), Augusta-Kliniken Bochum, Bergstr. 26, 44791 Bochum, Deutschland

**Keywords:** Todesangst, Operationsangst, Anästhesiekomplikationen, Postoperativer Verlauf, Death anxiety, Surgery anxiety, Complications of anesthesia, Postoperative course

## Abstract

In der Anästhesiologie wird man häufig mit Patienten konfrontiert, die unter perioperativen Ängsten und speziell der Angst vor dem Tod leiden, dies gilt jedoch nicht nur für die Anästhesiologie, sondern auch für die Psychiatrie und die Psychotherapie. Bislang ist die Literaturlage hierzu begrenzt, und daher werden diesem Übersichtsartikel die wichtigsten Arten von perioperativer Angst, diagnostische Aspekte sowie Risikofaktoren thematisiert. Anxiolytisch werden klassischerweise Benzodiazepine eingesetzt, in den letzten Jahren ist jedoch die präoperative Angst reduzierende Wirkung von z. B. supportiven Gesprächen, Akupunktur, Aromatherapie und Entspannungsverfahren stärker in den Fokus geraten, da Benzodiazepine unter anderen ein postoperatives Delir mit Zunahme von Morbidität und Mortalität fördern. Perioperative Ängste vor dem Tod sollten jedoch klinisch und wissenschaftlich verstärkt in den Blick genommen werden, um nicht nur die Patienten präoperativ besser versorgen, sondern auch um nachteilige Folgen im Verlauf von Operationen und danach reduzieren zu können.

## Abstract (en)

An important field of anesthesiology but also of psychiatry and psychotherapy, is perioperative anxiety and especially the fear of death. In this review article the most important types of anxiety in the individual phases before, during and after surgery are presented and diagnostic aspects as well as risk factors are discussed. Benzodiazepines can classically be used therapeutically here, but in recent years the preoperative anxiety-reducing effects of e.g., supporting talks, acupuncture, aroma therapy, and relaxation methods have come more into focus, because benzodiazepines promote postoperative delirium, which significantly increases morbidity and mortality. Perioperative fear of death should, however, be given greater clinical and scientific attention in order not only to have a better understanding and preoperative care of patients, but also to reduce adverse consequences during surgery and afterwards.

## Einleitung

Gemäß dem Statistischen Bundesamt (Nr. 437, 4/11/2020) wurden im Jahr 2019 mehr als 7 Mio. der über 18. Mio. stationär behandelten Patienten in Deutschland operiert. Es wird angenommen, dass zwischen 40 und 80 % der zu operierenden Patienten Ängste im Zusammenhang mit einer Operation entwickeln [12]. Dabei scheint es keine Rolle zu spielen, ob der operative Eingriff als Notfall oder als Elektiveingriff erfolgt. Vielmehr begründen sich diese im Kontext mit einer Operation stehenden Ängste aus einer Fülle von verschiedenen Faktoren. Ein höherer psychosozialer Stress bis hin zu einer Depression gilt u. a. als prädisponierender Faktor für die Entwicklung einer erhöhten Angst im Rahmen einer Anästhesie und vor einem operativen Eingriff. Daher wird diese Angst auch als präoperative Angst, im Englischen als „preoperative anxiety“, bezeichnet [11]. Die Fülle der Veröffentlichungen zur präoperativen Angst und zu den verschiedenen Interventionsmöglichkeiten unterstreicht die Bedeutung dieser Thematik, v. a. für die Bereiche der Anästhesie und Chirurgie. Aber auch in der alltäglichen Tätigkeit eines Psychiaters und Psychotherapeuten spielt Angst als Symptom und als ein psychopathologisches Merkmal verschiedener psychiatrischer Störungen eine besondere Rolle. Die sich daraus ableitenden, fachübergreifenden Überschneidungen zeigen sich nicht zuletzt in den etablierten psychotherapeutischen Interventionen (z. B. [54]).

Die Situation, sich einer Operation zu unterziehen, wird von vielen Menschen als eine existenzielle Grenzerfahrung betrachtet und erlebt, da sie uns an die „Grenzen unseres Seins“ stößt und unsere „Zerbrechlichkeit des Seins“ (nach dem Philosophen Karl Jaspers) aufzeigt. Es ist daher nicht verwunderlich, dass existenzielle Ängste v. a. in Form einer Angst vor dem Tod bei einer solchen Grenzerfahrung auftreten [25]. Die Ängste beziehen sich darauf, die Kontrolle über die eigene Existenz, den eigenen Körper, das eigene „Ich“ im Rahmen einer Allgemeinanästhesie zu verlieren und anderen ausgeliefert zu sein [42]. Welche Bedeutung konkret dabei die Angst vor dem Tod im Kontext perioperativer Ängste spielt, ist paradoxerweise nicht geklärt. Diese wird nicht explizit erfragt und bis dato nicht differenziert untersucht. Es finden sich in den vielfältigen Studien zu perioperativen Ängsten lediglich indirekte Hinweise, dass präoperative Patienten häufig eine Angst vor dem Tod, v. a. aber eine Angst vor dem Sterben empfinden.

## Perioperative Ängste

Angst ist zunächst eine grundlegende, ubiquitäre Basisemotion und eine biologisch determinierte physiologische Reaktion des Organismus auf bedrohliche und unkontrollierbare Gefahrensituationen (gemäß S3-Leitlinien Angststörungen). Als angemessene Angst übt sie eine Alarmfunktion bei bestehenden Gefahren für den Organismus aus und trägt damit zu seinem Schutz und Erhalt bei. Eine übermäßig gesteigerte unrealistische, inadäquate Angst geht hingegen als pathologische Angst mit deutlichen psychischen und körperlichen Funktionseinschränkungen einher. Der Übergang von einer zunächst physiologischen zu einer pathologischen Angst ist fließend. Hierbei ist auch noch nicht geklärt, warum es nur bei einem Teil der Menschen überhaupt dazu kommt.

Ängste, die in Verbindung mit einer Operation und Narkose auftreten, sind seit Langem im Fokus der klinischen Praxis und der Forschung in der Anästhesiologie. So konnte gezeigt werden, dass sowohl das Ausmaß der Angst als auch die Angstinhalte sich in den verschiedenen perioperativen Phasen im Kontext einer Operation deutlich unterscheiden [9]. Aus der Literatur ergeben sich Hinweise, dass es bei Patienten, die sich einer elektiven Operation unterziehen müssen, häufig zum Auftreten sowohl präoperativer als auch postoperativer Angst kommt. Dabei scheint die präoperative Angst ein sehr guter Prädiktor für das Auftreten und das Maß der postoperativen Angst zu sein [38]. Zudem wurde in zahlreichen Studien herausgefunden, dass eine erhöhte präoperative Angst den intra- und postoperativen Verlauf negativ beeinflusst [50]. Dies geht mit häufigerem Auftreten von intra- und postoperativen Komplikationen wie z. B. Aspiration, Übelkeit, Erbrechen [15] einher. Es konnte auch gezeigt werden, dass eine hohe präoperative Angst mit einem erhöhten und schwer zu beherrschenden postoperativen Schmerzerleben einhergeht [1]. Yang et al. konnten im Rahmen einer Metaanalyse von 33 Studien mit mehr als 53000 Patienten neben der präoperativen Angst weitere wichtige Prädiktoren herausarbeiten, die mit einem schlechteren postoperativen Schmerz-Coping einhergehen. So beeinflussen jüngeres Alter, weibliches Geschlecht, Nikotinkonsum, präoperative depressive Symptome, Schlafstörungen, hoher BMI sowie präoperativ schon bestehende Schmerzen den postoperativen Verlauf der Schmerzsymptomatik nachteilig [58]. Interessanterweise spielen die Art der Operation und der hierfür zugrunde liegende Anlass weniger eine Rolle, wie Costelloe et al. [8] in ihrer Metaanalyse mit mehr als 20 Studien zu persistierenden postoperativen Schmerzen schlussfolgerten. Angst und Depression in der präoperativen Phase erwiesen sich auch hier als wesentliche Faktoren, die postoperativ zu länger andauernden Schmerzen beitragen. Allerdings scheinen für die Ausprägung präoperativer Ängste auch individuelle Faktoren wie Alter, Geschlecht und Bildungsgrad eine Rolle zu spielen [12]. Bei genauer Betrachtung der Studien, die sich mit perioperativen Ängsten und der Anästhesieform befassten, fällt auf, dass sowohl bei Vollnarkosen als auch bei Regionalanästhesien Ängste auftreten. Einerseits wird berichtet, dass v. a. Patienten mit höherer präoperativer Ängstlichkeit häufiger eine Allgemeinanästhesie für sich auswählen [43], andererseits zeigen Patienten, die sich für eine Regionalanästhesie entscheiden, höhere präoperative Cortisol- und Angstwerte als jene, die sich einer Allgemeinanästhesie unterziehen. Zudem finden sich auch Berichte, die keinen Unterschied und keine direkte Beziehung zwischen präoperativer Angst und Art der Narkose konstatieren [40]. Es lässt sich daher nur spekulieren, ob sehr ängstliche Patienten im Sinne psychischer Abwehr eher zu einer Allgemeinanästhesie neigen, während weniger ängstliche Menschen die Regionalanästhesie präferieren. Hier würde dann physiologisch eine Stress-Angst-Reaktion vor dem chirurgischen Angriff nachvollziehbar eintreten. Für fundierte Aussagen hierzu bedarf es weiterer interdisziplinärer Forschung, zumal das Untersuchungskollektiv je nach Grunderkrankung und Operation heterogen ausfällt [52].

Bezüglich einer inhaltlichen Unterscheidung der Ängste in Abhängigkeit von den verschiedenen perioperativen Phasen ist es nachvollziehbar, dass die präoperative Phase für die Mehrheit der Betroffenen, die vorher keine Erfahrungen mit chirurgischen Eingriffen hatten, mit mehr Ängsten vergesellschaftet ist als bei denjenigen Patienten mit Vorerfahrung [47]. Die Angst vor dem „Unbekannten“ nach einer überstandenen Operation ist deutlich geringer, und andere Sorgen prägen den postoperativen Verlauf. Es wäre dabei die These aufzustellen, dass die Angst in der präoperativen Phase eine mehr anästhesiebezogene ist als die Angst in der postoperativen Phase, welche eher durch chirurgische Aspekte z. B. der Wundheilung gekennzeichnet ist. Eine Differenzierung zwischen den Ängsten in Bezug auf die Anästhesie, getrennt von den allgemeinen Operationsängsten, wird oft kontrovers diskutiert [12].

Präoperative Angst wird v. a. durch das Gefühl, einer Situation hilflos ausgeliefert zu sein, im Sinne eines Verlustes der Kontrolle bzw. Kontrollierbarkeit der Krankenhaussituation verstärkt. Hinzu tritt ein Schamgefühl, sich und den eigenen Körper unkontrolliert anderen gegenüber bloßzustellen („nudeness“ [35]). Wesentlich ist auch eine Angst vor der Anästhesie im Zusammenhang mit Sorgen, während der Narkose zu ersticken, während des operativen Eingriffs das Bewusstsein zu erlangen („awareness“) oder nicht mehr aus der Narkose aufzuwachen [12]. Sorgen bereitet auch das postoperative Delir (POD), welches v. a. ältere Patienten betrifft. Dieses ist nicht nur mit teilweise schwerwiegenden postoperativen kognitiven Dysfunktionen und psychosozialen Einschränkungen bei bis zu 50 % der betroffenen Patienten, sondern mit einer zeitweise bis zu 20fach erhöhten Mortalität verbunden [52].

Allerdings spielen auch präoperativ Sorgen bezüglich der Operation wie z. B. das Ausmaß des chirurgischen Eingriffs, die Ungewissheit über Operationsverlauf und Operationsergebnis, die Angst vor postoperativen Komplikationen und v. a. postoperativen Schmerzen für die betroffenen Patienten eine bedeutende Rolle [10].

Konkrete Angaben zu Häufigkeit und Intensität der „existenziellen Angst vor dem Tod“ in den perioperativen Phasen sind nur bedingt möglich, da nur wenige Studien dazu vorliegen.

Die Unterscheidung zwischen anästhesiologischer und chirurgischer Angst ist umstritten, da sie theoretischer Natur zu sein scheint, zumal aus der Sicht der Patienten Anästhesie und Operation häufig in denselben Zusammenhang gesetzt werden [23]. Dies könnte daher rühren, dass Patienten die Anästhesiologie nicht als eigenes medizinisches Fach wahrnehmen, obwohl nicht selten Ängste vor der Narkose klarer und häufiger formuliert werden als Sorgen und Angst vor der eigentlichen Operation [23]. Dies mutet irrational an, da man davon ausgehen könnte, dass z. B. eine kardiochirurgische Bypass-Operation, im Rahmen derer thorakotomiert, ein Herz-Kreislauf-Stillstand induziert wird und die Herz-Lungen-Maschine (HLM) die Arbeit von Lungen und Herz übernimmt, mehr Ängste bedingen müsste. Tatsächlich finden sich nur wenige Untersuchungen, bei denen die Operationsangst stärker als die anästhesiebezogene Angst ausfällt. Dies betrifft Operationen, die von den Patienten als schwere, teilweise den Körper verstümmelnde Eingriffe erlebt werden [22].

Ein wichtiger Aspekt scheint der Aufklärungs- und Informationszustand der Patienten zu sein [57]. Wiederholt konnte gezeigt werden, dass das Wissen um Zuständigkeiten und Kompetenzen des Anästhesisten häufig unzureichend ist [6]. Ein in der Literatur oft genannter Grund hierfür wird in den vielseitigen anästhesiologischen Aufgabenbereichen gesehen, denn Anästhesie bedeutet heutzutage Anästhesiologie, Intensivmedizin, Notfallmedizin und Schmerztherapie (AINS) [30]. Hinsichtlich der Rolle als „perioperativ Mediziner“ liegt der Fokus des Anästhesisten auf dem Prämedikationsgespräch. Einen hohen Stellenwert dabei hat das Bemühen, Ängste der Betroffenen zu reduzieren und Vertrauen zu schaffen [5]. Das dieser hohe Anspruch durch den ständigen Personalmangel und finanzielle Zwänge im Klinikalltag nicht immer zu realisieren ist, stellt nicht nur für die Anästhesiologie, sondern für die gesamte Medizin eine Herausforderung dar.

Die Forderung nach mehr Aufklärung und Information über die vielseitigen Aufgaben des Anästhesisten nicht nur im Rahmen eines operativen Eingriffs erscheint daher nachvollziehbar [30]. Sie setzt aber auch eine differenzierte Untersuchung der Angstinhalte in den verschiedenen Segmenten voraus [12]. Die Erforschung von spezifischen, auch individuell unterschiedlich geprägten perioperativen Ängsten ist von großer klinischer Bedeutung. Dadurch ließe sich z. B. bei den von starken präoperativen Ängsten betroffenen Patienten rechtzeitig ein differenzielles Behandlungsprozedere zur Verbesserung des postoperativen Outcome erarbeiten [45].

### Behandlungs- und Interventionsmöglichkeiten perioperativer Ängste

Die erste effektive Intervention hinsichtlich einer vorhandenen präoperativen Angst stellt das Prämedikationsgespräch dar. In Studien fand sich wiederholt, dass bestehende Ängste durch die von den Anästhesisten durchgeführte Prämedikationsvisite deutlich vermindert werden [27, 46]. Interessanterweise scheint der Zeitpunkt der Prämedikationsvisite wichtig zu sein, zumal Klopfenstein et al. [27] zeigen konnten, dass ein Termin 2 Wochen vor dem geplanten chirurgischen Eingriff hierfür effektiver ist als am Vorabend der Operation. Der Einsatz von Medientechnologien (z. B. Video, virtuelle Realität, Anästhesie-Service-Plattformen) kann zur Informationsvermittlung dienen. Sie können zur Operationsvorbereitung wesentlich dazu beitragen, auf die Fragen, Unsicherheiten und Ängste der Patienten entsprechend den individuellen Gegebenheiten wie Alter, Bildung, Geschlecht und Art der Operation einzugehen. Dadurch lassen sich präoperative Ängste und konsekutiv postoperative Komplikationen, v. a. postoperative Schmerzen, die insgesamt einen wesentlichen Einfluss auf die Morbidität haben, deutlich reduzieren [51]. In Analogie zu den verhaltenstherapeutischen In-vivo-Expositionsverfahren können die betroffenen Patienten auch mittels digitaler Simulation (VR-Brillen) mit den angstbesetzten Stimuli (Situationen wie z. B. der OP) konfrontiert werden und in der jeweiligen virtuellen Umgebung dann angstdesensibilisierend reagieren. Hierbei können psychopathologisch relevante Reaktionen dann analysiert und bestenfalls modifiziert werden [56].

Die zusätzliche chirurgische Aufklärung sowie psychoedukative Gespräche – auch seitens des Pflegepersonals – mit dem Ziel, den Patienten über den Eingriff und die damit zusammenhängenden Risiken zu informieren, haben ebenfalls einen günstigen Einfluss auf die perioperative Angst, wie eine aktuelle Metaanalyse anhand 9 Studien mit fast 450 Patienten zeigen konnte [45]. Fehlende Informationen, ungünstige Aufklärungsbedingungen wie z. B. Zeitdruck, aber auch eine ablehnende Haltung des Patienten, sich mit den möglichen negativen Folgen einer anstehenden Operation zu beschäftigen, können Ängste verstärken [13].

Eine Vielzahl von Studien konnte die Effektivität kognitiv-verhaltenstherapeutischer Interventionen bezüglich Angstreduktion bei unterschiedlichen somatisch erkrankten Patientengruppen aufzeigen [9, 16, 34, 54]. Die einfach von Patienten erlernbare progressive Muskelrelaxation nach Jacobson kann nicht nur zu einer Entspannung und Verminderung perioperativer Angst führen, sondern auch den Gebrauch von Opioiden zur Kupierung postoperativer Schmerzen reduzieren [16]. Studien konnten zudem zeigen, dass verschiedene kurze Interventionen wie z. B. achtsamkeitsbasierte kognitive Techniken [35], Akupunktur [55] oder Hypnose [2] ebenfalls zur deutlichen Reduktion von präoperativer Angst effektiv eingesetzt werden können. Besonders interessant sowohl hinsichtlich der Praktikabilität und Effizienz als auch patientenbezogener Akzeptanz und Verträglichkeit sind komplementäre Interventionsstrategien wie die Aroma- [20] oder imaginative Gestalt‑, Kunst- und Musiktherapie [1]. Die umfangreiche Literatur täuscht allerdings über die Tatsache hinweg, dass es nur wenige kontrollierte Untersuchungen mit teilweise auch widersprüchlichen Ergebnissen bezüglich der Wirksamkeit dieser Verfahren gibt [53]. Dabei scheint z. B. der positive Einfluss des Hörens der Lieblingsmusik stärker auf die präoperative Angst zu wirken als auf das postoperative Schmerzerleben [53]. Zudem scheinen Frauen stärker als Männer von musiktherapeutischen Angeboten und einer Aromatherapie in Form von verschiedenen Ölen (z. B. Lavendel‑, Zitrus- oder Rosenöl zur Inhalation oder zur Massage) anzusprechen [28]. Zuletzt konnte auch mehrfach gezeigt werden, dass die Durchführung von Yoga an wenigen Tagen vor der Operation perioperative Ängste reduzieren kann [3].

Der Einsatz von Benzodiazepinen (z. B. Midazolam oder Triazolam) bei präoperativen Ängsten v. a. bei älteren Patienten wird trotz unbestrittener Wirksamkeit kontrovers diskutiert. Grund dafür sind schwere unerwünschte Arzneimittelwirkungen wie Atemdepression, häufigeres postoperatives Delir (POD), kognitive Beeinträchtigungen, anterograde Amnesien und paradoxe Reaktionen [29]. Das Auftreten eines durch Benzodiazepine gefördertes POD ist mit einer erhöhten postoperativen Morbidität und Mortalität assoziiert [52]. Daher erfolgt gerade bei älteren Patienten mit kognitiven Einschränkungen oder demenziellen Syndromen mittlerweile kein routinemäßiger Einsatz von Benzodiazepinen. Diese sollten lediglich bei strenger Indikation mit vordiagnostizierter Angststörung eine mögliche Behandlungsoption sein [44]. Melatonin erweist sich nach einer Metaanalyse von Madsen et al. [33] unter Einschluss von 27 kontrollierten Studien als deutlich verträglicher als die Benzodiazepine und gleich wirksam hinsichtlich der Reduktion präoperativer Ängste. Auch in Bezug auf postoperative Ängste zeigt Melatonin eine zwar geringere, trotzdem einem Placebo überlegene Wirksamkeit [33]. Auch für den α_2_-Adrenorezeptor-Agonist Clonidin [19] sowie für das als Antiepileptikum bekannte Pregabalin gibt es Berichte über gute anxiolytische Wirkung bei präoperativen Ängsten. Zudem gewinnen beide letztgenannten Substanzen in jüngster Zeit auch im Rahmen des perioperativen Settings zusätzlich sschmerztherapeutische Bedeutung. Durch ihre opioideinsparenden Effekte sind sie gegen drohende Chronifizierung von Schmerzen und Ängsten hilfreich (z. B. [24]). Insgesamt sind die Ergebnisse hier jedoch inkonsistent, sodass eine abschließende Beurteilung noch schwierig ist [48].

### Angst vor dem Tod

Konsistent hingegen ist der Befund, dass Patienten mit einer schweren somatischen Erkrankung und infauster Prognose eine stärkere Angst vor dem Tod haben, je mehr psychosozialer Stress vorliegt und zudem eine psychiatrische Komorbidität v. a. in Form einer Depression oder Angsterkrankung besteht [7]. Bei der Sichtung der Literatur zeigt sich aber insgesamt, dass die Angst vor dem Tod sowohl bei schwer somatisch erkrankten als auch bei psychiatrischen Patienten empirisch wenig untersucht ist [21], obwohl es an Skalen und Fragebogen zur Erfassung der Angst vor dem Tod nicht mangelt (z. B. Übersicht [59]). Vor allem die Studien im Rahmen der „Terror-Management-Theorie“ (TMT) haben in den letzten Jahren das Forschungsfeld hierzu dominiert. Die TMT [18] befasst sich mit den Reaktionsweisen (Management) der Menschen im Umgang mit der Angst vor dem Tod und dem Bewusstwerden der eigenen Endlichkeit (Terror). Sie postuliert, dass Selbstwert und kulturelle Weltanschauung effektive Coping-Strategien hinsichtlich der sog. mortalitätssalienzbedingter Angst (Bewusstwerden der eigenen Sterblichkeit) darstellen. Die der TMT zugrunde liegenden Untersuchungen [32] basieren hauptsächlich auf Experimenten, welche bei Militärpersonal und Studenten unterschiedlicher Nationalität die Mortalitätssalienz hervorriefen und nach Induktion dieser Einstellungs- und Verhaltensänderungen in Form von Verteidigung des eigenen kulturellen Weltbildes und des persönlichen Wertes gegenüber einer Bedrohung von außen bedingten. Zusammengefasst ergaben diese allgemeinen Untersuchungen zur Angst vor dem Tod bei unterschiedlichen gesunden Normpopulationen wie z. B. Krankenschwestern, Studenten und anderen im Gesundheitswesen Tätigen, dass Frauen häufiger als Männer über eine Angst vor dem Tod berichten. Ein höherer Bildungsgrad sowie ein guter sozioökonomischer Status und ein guter körperlicher Zustand gehen mit einer niedrigen Rate von Todesangst einher. Altersunterschiede ließen sich nicht durchgehend aufzeigen, ebenso wenig, dass ein tieferer Glaube nicht zwangsläufig eine niedrige Rate von Angst vor dem Tod bedingt. Psychische Probleme hingegen gehen mit einer höheren Angst vor dem Tod einher (Übersicht: [21]).

Erste eigene Untersuchungen mittels des „Bochumer-Fragebogen zur Erfassung der Angst vor dem Tod und der Einstellung zum Tod“ (BOFRETTA) [25] konnten zeigen, dass Patienten mit einer schweren psychischen Erkrankung wie Depression oder Schizophrenie eine stärkere Angst vor dem Tod als psychisch gesunden Menschen aufweisen [37]. Während die Angst vor dem Tod zwischen den beiden Patientengruppen nahezu gleich hoch war, wiesen die Patienten mit einer Erkrankung aus dem schizophrenen Formenkreis eine deutliche negative und somit nichtsouveräne Einstellung zum Tod auf. Patienten mit einer depressiven Erkrankung erleben sich im Kontext ihrer psychischen Belastung und Depressivität in einer deutlichen Sinnkrise, was wiederum mit einer erhöhten Angst vor dem Tod assoziiert ist. Dabei können Persönlichkeitsdimensionen wie emotionale Labilität und ein verändertes Selbstwertgefühl das Ausmaß der Angst vor dem Tod beeinflussen [36].

Bei schwer somatisch erkrankten Patienten konnten wir mittels des BOFRETTA zeigen, dass Patienten mit einer amyotrophen Lateralsklerose (ALS) ähnlich wie ihre pflegenden Angehörigen eine unerwartet geringe Angst vor und eine weniger problematische Einstellung zum Tod aufweisen [17]. Ebenso zeigten an multipler Sklerose (MS) erkrankte Patienten eine weniger problematische Einstellung zum und niedrig einzustufende Angst vor dem Tod [14]. Auch Soleimani et al. [49] kommen in ihrer Metaanalyse der Studien zu Angst vor dem Tod bei Patienten mit einer Krebserkrankung zu dem Ergebnis, dass die Angst vor dem Tod bei diesen schwer Erkrankten lediglich in moderater Ausprägung vorliegt. Als mögliche Erklärungen hierbei sind denkbar, dass eine Verdrängung der Todesthematik im Sinne einer Abwehrstrategie oder die Einstellung, den Tod als eine Erlösung vom Leiden zu betrachten, vorliegt. Regionale, soziokulturelle Unterschiede (Asien > Europa > Nordamerika), Art der Krebserkrankung (Brustkrebs), Geschlecht (weiblich) und Beziehungsstatus (verheiratet sein) erwiesen sich dabei als negative Einflussfaktoren auf die Angst vor dem Tod [49]. Ob hierbei Persönlichkeitsdimensionen, Lebenssinn oder krankheitsspezifische Faktoren als Angstpuffer bzw. als Trigger der Angst vor dem Tod wirken, muss in zukünftigen Studien mit körperlich schwer erkrankten Patienten weiter untersucht werden.

## Präoperative Angst vor dem Tod bei chirurgischen Eingriffen

Welche Bedeutung die Angst vor dem Tod und die Einstellung zum Tod im Zusammenhang mit perioperativen Ängsten haben, ist paradoxerweise nicht geklärt, da diese auch nicht differenziert genug untersucht wurden. Es finden sich in den vielfältigen Studien, in denen präoperative Ängste allgemein meistens mittels der Visual Analogue Scale for Anxiety (VAS [26]), des „State and Trait Anxiety Inventory“ (STAI-I/II Inventar, [31]) und der „Amsterdam Preoperative Anxiety and Information Scale“ (APAIS, [4]) erfasst wurden, lediglich indirekte Hinweise, dass präoperative Patienten häufig eine Angst vor dem Tod bzw. Sterben empfinden. Die Skala APAIS dürfte aufgrund seiner Kürze (< 5 min für 6 Fragen) und seiner Spezifität für präoperative Ängste für die Praxis am besten geeignet sein. Diese regelmäßig anzuwenden, zusammen mit den mittlerweile bereits im Studium gelernten Techniken der ärztlichen Gesprächsführung, wäre wünschenswert. Fitzgerald und Elder [13] fanden bei der Befragung von 387 zufällig rekrutierten Patienten im Rahmen ihres Anästhesieaufklärungstermins vor der geplanten Operation, dass bei der Hälfte (50,6 %) dieser die Angst vor dem Tod am häufigsten genannt wurde. Auch Nigussie et al. [41], die mittels STAI-I/II präoperative Ängste bei 239 chirurgischen Patienten untersuchten, berichten über das Bestehen einer Angst vor dem Tod bei über 38 %, gefolgt vom Vorhandensein einer diffusen und unspezifischen Angst bei immerhin 24,3 % der Patienten. Interessant bei dieser Untersuchung war auch der Befund, dass fast 80 % der befragten Patienten angaben, dass ihr religiöser Glaube und das Sprechen über die Ängste mit anderen Patienten für sie wichtige Bewältigungsstrategien zur Angstreduktion darstellten [41]. Eine aktuelle Untersuchung von 117 muslimischen Patienten vor einem plastisch-chirurgischen Eingriff konnte zeigen, dass diejenigen mit einer stärkeren Religiosität in Form der häufigeren Ausübung der religiösen Rituale deutlich weniger präoperative Angst aufwiesen als die weniger religiösen Patienten [39]. Dies kann als Hinweis gesehen werden, dass Religiosität zur Reduktion von präoperativen Ängsten beitragen kann.

Das Wissen um die Bedeutung und Auswirkungen der Angst vor dem Tod im Allgemeinen und speziell im Kontext eines anästhesiologisch-chirurgischen Eingriffs zu Behandlung und Heilung eines körperlichen Leidens kann jedoch eine Möglichkeit darstellen, Menschen in dieser Situation besser zu verstehen. Basierend darauf sollten differenzierte und individualisierte Hilfs- und Behandlungsangebote erarbeitet werden, welche als Angstpuffer dienen und somit konsekutiv negative postoperative Komplikationen wie z. B. postoperative Schmerzen, Delir oder Wundheilungsstörungen verhindern.

## Fazit für die Praxis

- Perioperative und insbesondere existenzielle Ängste sollten nicht nur üblicherweise im Rahmen der Prämedikationsvisite durch die Anästhesisten thematisiert werden, sondern zunehmend auch im Fokus des chirurgischen Aufklärungsgesprächs stehen.
- Psychiatrisch vorerkrankte Patienten und Patienten ohne operative Vorerfahrungen weisen höhere, intensivere und länger andauernde präoperative Ängste auf.
- Diese Ängste sollten in Form einer fachübergreifenden Fallbesprechung thematisiert und eine frühzeitige Interventionsbehandlung sollte eingeleitet werden, um dadurch einen besseren postoperativen Verlauf zu erreichen.
